# Supplementary material for: Targeted mutations in IFNα2 improve its antiviral activity against various viruses
Source: mBio. 2023 Oct 24;14(6):e02357-23. doi: 10.1128/mbio.02357-23 (PMC10746204; doi:10.1128/mbio.02357-23)
Supplement: Fig. S1 — Sequence alignment of IFNα2, IFNα6, IFNα14, and IFNα2 mutants. [file mbio.02357-23-s0001.pdf]

Supp. Fig. 1

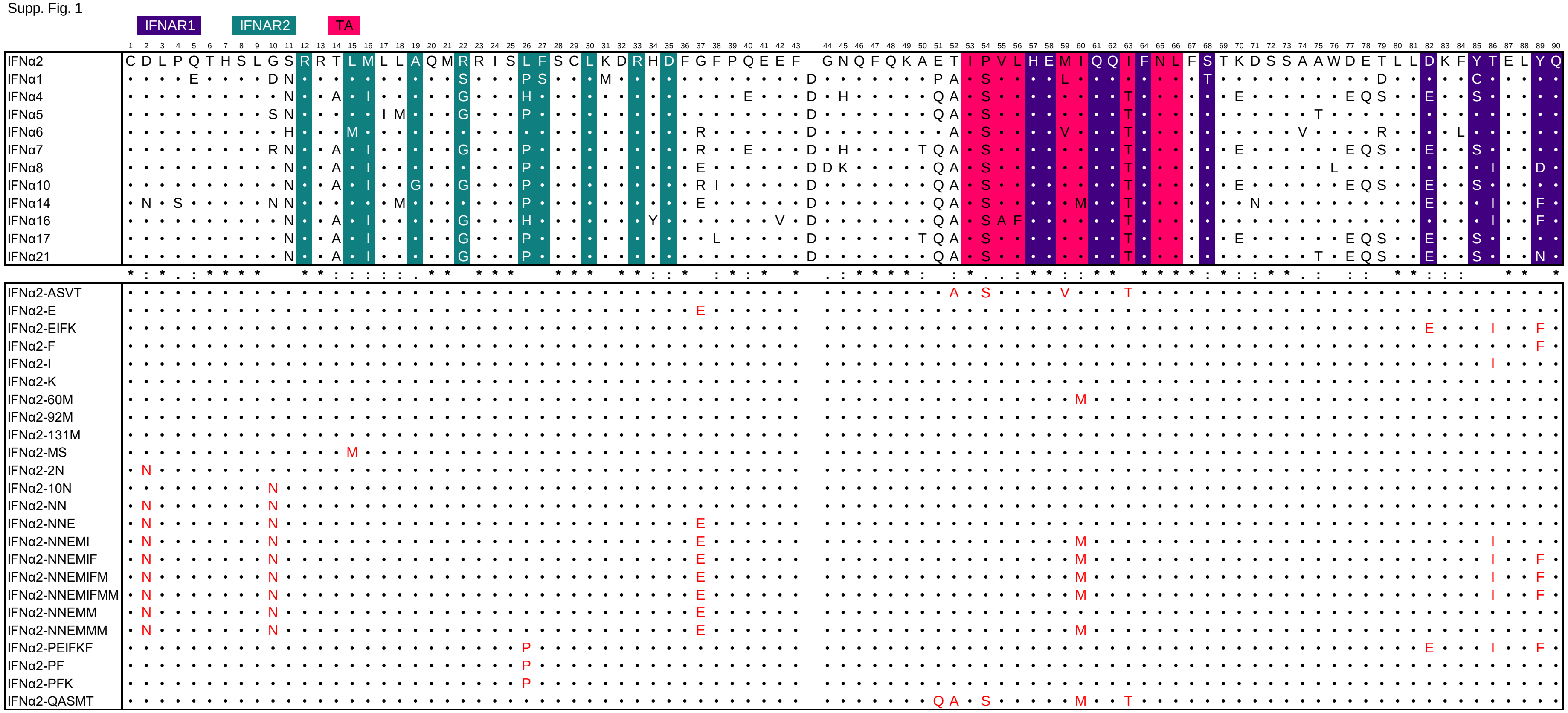

IFNAR1
IFNAR2
TA
IFNα2 C D L P Q T H S L G S R R T L M L L A Q M R R I S L F S C L K D R H D F G F P Q E E F G N Q F Q K A E T I P V L H E M I Q Q I F N L F S T K D S S A A W D E T L L D K F Y T E L Y Q
IFNα1
IFNα4
IFNα5
IFNα6
IFNα7
IFNα8
IFNα10
IFNα14
IFNα16
IFNα17
IFNα21
IFNα2-ASVT
IFNα2-E
IFNα2-EIFK
IFNα2-F
IFNα2-I
IFNα2-K
IFNα2-60M
IFNα2-92M
IFNα2-131M
IFNα2-MS
IFNα2-2N
IFNα2-10N
IFNα2-NN
IFNα2-NNE
IFNα2-NNEMI
IFNα2-NNEMIF
IFNα2-NNEMIFM
IFNα2-NNEMIFMM
IFNα2-NNEMM
IFNα2-NNEMMM
IFNα2-PEIFKF
IFNα2-PF
IFNα2-PFK
IFNα2-QASMT

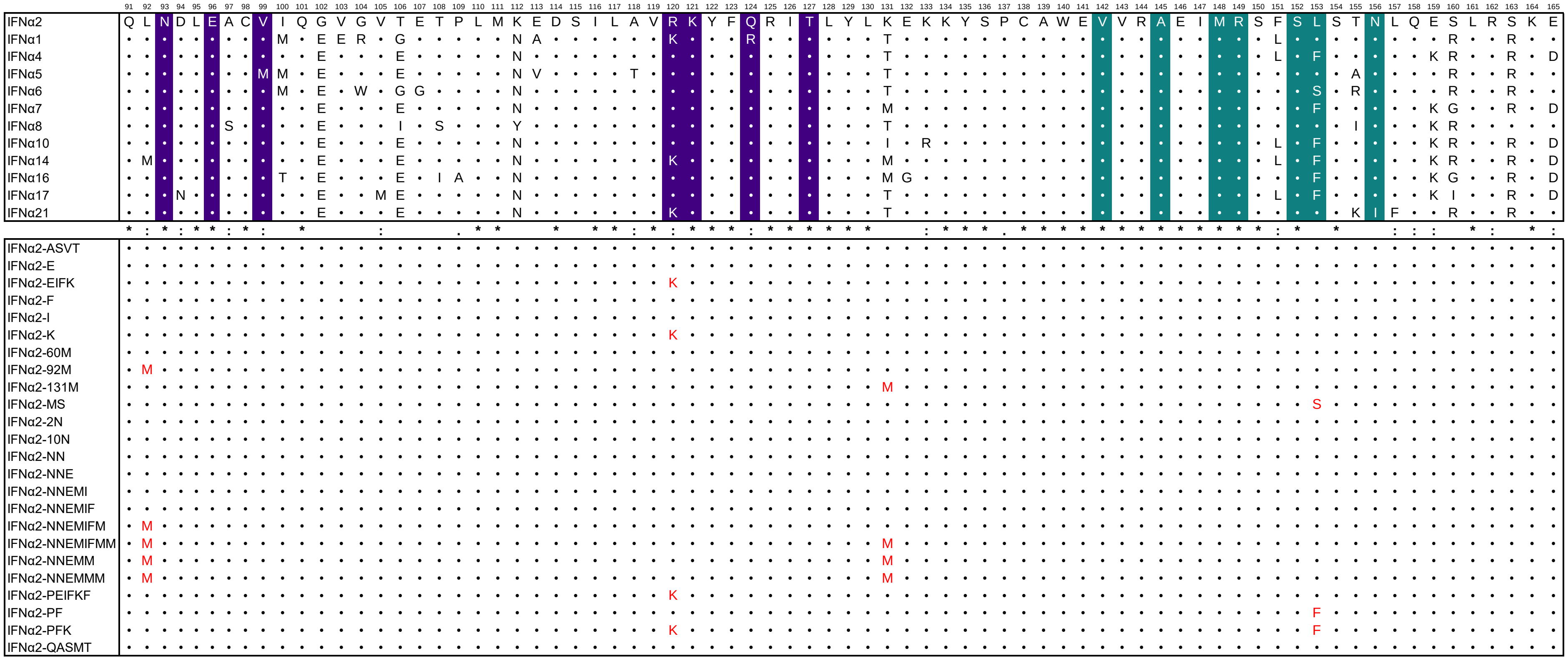

91–165
IFNα2 Q L N D L E A C V I Q G V G V T E T P L M K E D S I L A V R K Y F Q R I T L Y L K E K K Y S P C A W E V V R A E I M R S F S L S T N L Q E S L R S K E
IFNα1
IFNα4
IFNα5
IFNα6
IFNα7
IFNα8
IFNα10
IFNα14
IFNα16
IFNα17
IFNα21
IFNα2-ASVT
IFNα2-E
IFNα2-EIFK
IFNα2-F
IFNα2-I
IFNα2-K
IFNα2-60M
IFNα2-92M
IFNα2-131M
IFNα2-MS
IFNα2-2N
IFNα2-10N
IFNα2-NN
IFNα2-NNE
IFNα2-NNEMI
IFNα2-NNEMIF
IFNα2-NNEMIFM
IFNα2-NNEMIFMM
IFNα2-NNEMM
IFNα2-NNEMMM
IFNα2-PEIFKF
IFNα2-PF
IFNα2-PFK
IFNα2-QASMT

Supp. Figure 1: Sequence alignment of IFNα2, IFNα6, IFNα14, and IFNα2-mutants

Sequence alignment of human IFNα subtypes. Residues facing IFNAR1 are highlighted in purple, IFNAR2 binding sites are highlighted in teal, tunable anchor residues are depicted in pink. Sequences of IFNα2-mutants show induced amino acid mutations in red. Identical positions within the alignment are shown by '*'; conserved substitutions by ':', and semi-conserved substitutions by '.'.
