# Supplementary material for: Targeted mutations in IFNα2 improve its antiviral activity against various viruses
Source: mBio. 2023 Oct 24;14(6):e02357-23. doi: 10.1128/mbio.02357-23 (PMC10746204; doi:10.1128/mbio.02357-23)
Supplement: Fig. S2 — Dose-response analysis for the half maximal inhibitory concentrations. [file mbio.02357-23-s0002.pdf]

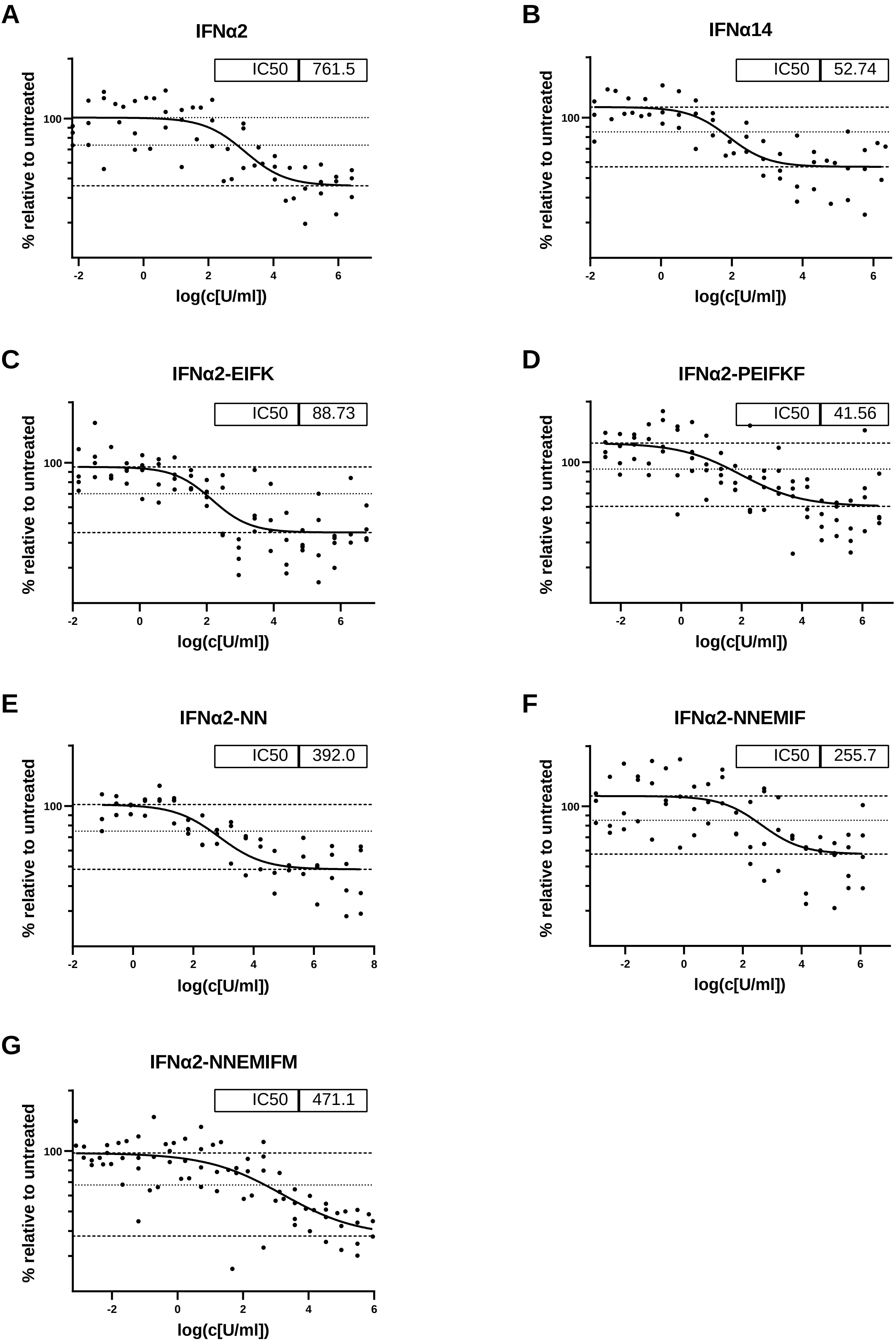
A
IFNα2
IC50
761.5
% relative to untreated
100
-2
0
2
4
6
log(c[U/ml])
B
IFNα14
IC50
52.74
% relative to untreated
100
-2
0
2
4
6
log(c[U/ml])
C
IFNα2-EIFK
IC50
88.73
% relative to untreated
100
-2
0
2
4
6
log(c[U/ml])
D
IFNα2-PEIFKF
IC50
41.56
% relative to untreated
100
-2
0
2
4
6
log(c[U/ml])
E
IFNα2-NN
IC50
392.0
% relative to untreated
100
-2
0
2
4
6
8
log(c[U/ml])
F
IFNα2-NNEMIF
IC50
255.7
% relative to untreated
100
-2
0
2
4
6
log(c[U/ml])
G
IFNα2-NNEMIFM
IC50
471.1
% relative to untreated
100
-2
0
2
4
6
log(c[U/ml])

Supp. Figure 2: Dose-response analysis for the half maximal inhibitory concentrations (IC_{50}). (A-G) TZM-bl cells were infected with a R5-HIV-$1_{NL4-3-IRES-Ren}$ reporter virus at a MOI of 0.02. A 3-fold dilution of the designated IFNs and IFNα2-mutants were performed starting with a 1:2 dilution in the first well. To determine the IC_{50} values, luciferase activity was measured 3 dpi and dose-response analyses were performed.
