# Supplementary material for: Targeted mutations in IFNα2 improve its antiviral activity against various viruses
Source: mBio. 2023 Oct 24;14(6):e02357-23. doi: 10.1128/mbio.02357-23 (PMC10746204; doi:10.1128/mbio.02357-23)
Supplement: Table S1 — Sequence entropies computed for the multiple sequence alignment. [file mbio.02357-23-s0003.pdf]

Supp. Table 1: Sequence entropies computed for the multiple sequence alignment

pos	cons	H	sas	sec	neighbor
1	C	0	624124	C	0
2	D	4,13817E+14	1142569	T	0
3	L	0	665734	T	0
4	P	4,13817E+14	487138	T	0
5	Q	4,13817E+14	1565962	T	0
6	T	0	121167	C	0
7	H	0	1455548	C	0
8	S	0	698582	C	0
9	L	0	565781	H	0
10	G	1,58496E+14	7476	H	0
11	N	8,16689E+14	228	H	0
12	R	0	823688	H	0
13	R	0	931374	H	0
14	-	9,79869E+14	0	H	0
15	L	4,13817E+14	104282	H	0
16	-	9,79869E+14	926385	H	2
17	L	4,13817E+14	2287	H	0
18	L	6,50022E+14	0	H	0
19	A	4,13817E+14	22669	H	2
20	Q	0	871831	H	0
21	M	0	0	H	0
22	-	1,28067E+14	1141827	T	0
23	R	0	1065418	T	0
24	I	0	528058	T	0
25	S	0	550688	T	2
26	P	1,25163E+14	423078	G	2
27	F	4,13817E+14	1610515	G	2
28	S	0	697151	G	2
29	C	0	1238	G	2
30	L	0	863875	T	2
31	K	4,13817E+14	1953719	T	0
32	D	0	164724	T	0
33	R	0	1128412	T	2
34	H	4,13817E+14	898179	C	2
35	D	4,13817E+14	954864	C	2
36	F	0	209017	C	2
37	-	1,38443E+13	430718	C	0
38	F	8,16689E+14	22883	C	0
39	P	0	247329	T	0
40	Q	6,50022E+14	706463	T	0
41	E	0	1610783	T	0
42	E	4,13817E+14	430615	T	0
43	F	0	7628	T	0
44	G	4,13817E+14	398208	T	0
45	N	1,04085E+14	1153013	T	0
46	Q	0	727441	T	0
47	F	0	13593	T	0
48	Q	0	902356	T	0
49	K	0	1461736	T	0
50	A	6,50022E+14	131605	T	0
51	Q	1,04085E+14	130388	T	0
52	A	4,13817E+14	0	H	0
53	I	0	97088	H	0
54	S	4,13817E+14	122556	H	0
55	V	4,13817E+14	0	H	0
56	L	4,13817E+14	30165	H	0
57	H	0	336365	H	0
58	E	0	675471	H	1
59	M	8,16689E+14	0	H	0
60	I	4,13817E+14	2465	H	0
61	Q	0	698161	H	1
62	Q	0	541153	H	1
63	T	6,50022E+14	0	H	0
64	F	0	399002	H	1
65	N	0	772755	H	1
66	L	0	127108	H	1
67	F	0	0	H	0
68	S	4,13817E+14	486082	H	0
69	T	0	38746	C	0
70	K	9,18296E+13	1718589	H	0
71	D	4,13817E+14	383267	H	0
72	S	0	0	H	0
73	S	0	315424	H	0
74	A	4,13817E+14	897907	H	0
75	A	6,50022E+14	247931	H	0
76	W	4,13817E+14	54574	C	0
77	-	9,79869E+14	542835	C	0
78	-	9,79869E+14	1184892	H	0
79	-	1,65002E+14	940086	H	0
80	L	0	400192	H	0
81	L	0	0	H	0
82	-	1	808129	H	1
83	K	4,13817E+14	1041786	H	1
84	F	4,13817E+14	0	H	0
85	-	1,32501E+14	407923	H	1
86	T	8,11278E+14	894488	H	1
87	E	0	72669	H	0
88	L	0	0	H	0
89	Y	1,4183E+14	1427135	H	1
90	Q	0	827473	H	1
91	Q	0	19422	H	0
92	L	4,13817E+14	86829	H	1
93	N	0	859193	H	1
94	D	4,13817E+14	359655	H	0

Pos	alignment position
cons	consensus sequence
H	entropy (0 = conserved)
sas	solvent accessible surface (0.0 = completely buried in protein)
sec	secondary structure (C = coil, T = turn, H = helix)
neighbor	0 = not neighbor of a receptor chain
	1 = neighbor or receptor 1
	2 = neighbor of receptor 2 (0.6 nm distance between a Cα atom of IFNα and any atom of receptor)

95	L	0	134969	H	0
96	E	0	712545	H	0
97	A	4,13817E+14	218105	H	0
98	C	0	39046	H	0
99	V	4,13817E+14	540636	H	0
100	-	1,28067E+14	1111716	H	0
101	Q	0	863368	C	0
102	E	4,13817E+14	674241	T	0
103	V	4,13817E+14	498685	T	0
104	G	8,16689E+14	865	T	0
105	V	4,13817E+14	600148	T	0
106	E	1,4183E+14	817458	T	0
107	E	8,16689E+14	1761211	C	0
108	T	8,16689E+14	24937	C	0
109	P	4,13817E+14	905788	C	0
110	L	0	1435513	H	0
111	M	0	784509	H	0
112	N	8,16689E+14	351309	H	0
113	E	1,04085E+14	77991	H	0
114	D	0	634249	H	0
115	S	4,13817E+14	0	H	0
116	I	0	299857	H	0
117	L	0	704878	H	0
118	A	4,13817E+14	89007	H	0
119	V	0	0	H	0
120	R	8,11278E+14	1129832	H	1
121	K	0	1347045	H	1
122	Y	0	62849	H	0
123	F	0	48848	H	1
124	Q	4,13817E+14	921666	H	1
125	R	0	1101917	H	0
126	I	0	0	H	0
127	T	0	410212	H	0
128	L	0	97006	H	0
129	Y	0	68979	H	0
130	L	0	4	H	0
131	-	1,5511E+14	1213015	H	0
132	E	4,13817E+14	942008	H	0
133	K	4,13817E+14	639846	H	0
134	K	0	1835645	T	0
135	Y	0	495811	T	0
136	S	0	310905	T	0
137	P	4,13817E+14	1011925	H	0
138	C	0	233949	H	0
139	A	0	0	H	0
140	W	0	9769	H	0
141	E	0	21769	H	2
142	V	0	8964	H	2
143	V	0	0	H	0
144	R	0	10212	H	0
145	A	0	326029	H	2
146	E	0	129788	H	2
147	I	0	0	H	0
148	M	0	461301	H	2
149	R	0	1663697	H	2
150	S	0	0	H	0
151	-	9,79869E+14	0	H	0
152	S	0	279439	H	2
153	-	1,32501E+14	367331	H	2
154	S	0	0	H	0
155	T	1,58496E+14	19481	H	0
156	N	4,13817E+14	507584	T	2
157	L	4,13817E+14	160974	T	0
158	Q	4,13817E+14	703268	T	0
159	-	9,79869E+14	793314	T	2
160	R	1,4183E+14	61768	T	2
161	L	0	956082	T	2
162	R	4,13817E+14	2144231	C	2
163	R	6,50022E+14	8851	C	2
164	K	0	1756403	C	2
165	-	1	2451069	C	2
